# A literature review of contacting force measurement methods for pedestrian crowds

**DOI:** 10.1016/j.heliyon.2024.e39755

**Published:** 2024-10-23

**Authors:** Rongyong Zhao, Arifur Rahman, Bingyu Wei, Cuiling Li, Yunlong Ma, Yuxing Cai, Lingchen Han

**Affiliations:** School of Electronic and Information Engineering, Tongji University, Shanghai, 201804, China

**Keywords:** Pedestrian contact force, Crowd dynamics, Pressure measurement sensor, Motion capture system, Slope micro-road network, Numerical simulation, Pedestrian safety

## Abstract

This article reviewed state-of-the-art achievements in pedestrian contacting force measurement as a hotspot survey closer to ground truth supporting pedestrian dynamics in mass-gathering environments. It analyzed different forces acting on pedestrian bodies, including normal external forces, self-driven forces, abnormal external forces, and pedestrian motion constraint forces from other obstacles, besides the crowding posture on the force distribution. This review covered main methodologies: sophisticated pressure sensors, modern technology for pedestrian motion-capturing systems, and advanced numerical simulations. Further, this paper summarized key findings from recent studies related to pedestrian contacting or crowding forces. It was found that despite significant advances, study achievements are mainly limited to different crowding postures, such as experiments regarding controlled environments in flat areas, indoor corridors, staircases, and competitive evacuation drills. Lack of sufficient sensor-based body measurements and contact force measurements on slop roads was analyzed. Finally, future research outlook was outlined, including planned experiments in highly crowded environments.

## 1 Introduction

With the rapid development of the global economy and the continuous growth of the population, urbanization is accelerating at an unprecedented pace. Consequently, the mortality rate associated with crowd-related disasters has significantly increased, particularly in mass gatherings [1]. Each year, crowd disasters lead to many deaths and injuries globally. Recently, stampede killed at least 10 students in Cameroon [2]. In 2023, a mass stampede occurred in Brazzaville, the capital of the Republic of the Congo. This incident happened on the last day of a military recruitment event at the Michel d'Ornano Stadium, resulting at least of 32 deaths and 144 injuries [3]. Another devastating incident occurred during the Halloween celebrations in 2022, where over 158 people lost their lives due to a crowd crush in one of Seoul's popular nightlife districts [4]. A tragic event of a crowd crush occurred on Mount Meron, Israel, during the yearly pilgrimage in 2021 to the tomb of Rabbi Shimon bar Yochai on the Jewish festival of Lag BaOmer. It is estimated that forty-five males of all ages lost their lives, while another 150 individuals sustained injuries [5]. The annual Hajj trip in Mina, Mecca, Saudi Arabia, on September 24, 2015, led to an awful crowd crush that caused the death of over 2000 individuals. Many of them were covered or crushed, making it the deadliest Hajj tragedy ever recorded [6]. Understanding pedestrian dynamics is necessary to ensure crowd safety and efficiently handle huge crowds [7]. High-density events, such as religious gatherings, sporting events, concerts, and emergency evacuations, pose increasing challenges due to the complex interactions and dynamics among individuals [8].

Accurately measuring pedestrian contact forces is crucial for predicting crowd dynamics, engineering more secure public areas, and avoiding accidents. Physical contact between people occurs in dense crowds, leading to irregular crowd disturbance flows [9]. Both empirical data and simulation studies demonstrate that current research methodologies enable the prediction and manipulation of pedestrian behavior in various cultural contexts, thereby enhancing the safety and efficiency of crowded spaces. Additionally, contact forces are particularly critical in restricted areas of pedestrian trajectories, where they play a significant role in crowd dynamics, as observed in earthquake scenarios [10]. In hazardous situations, pedestrians are often subjected to forceful physical interactions with others, which can result in a loss of balance. Once an individual loses balance due to sudden contact, regaining stability becomes increasingly difficult, often leading to accidents [11]. This disturbance can lead to a chain reaction or cascade effect, culminating in stampedes [12]. In pedestrian-related accidents, the transfer of force and momentum between individuals plays a crucial role in the outcome of such events [13].

Higher levels of competitiveness have been shown to generate greater contact forces, rapid crowd movement, and increased stress near exit locations [11,14]. These findings suggest that social and psychological variables should be added to pedestrian dynamics models for describing and simulating actual behaviors during evacuations. Obstacles and unique architectural layouts create remarkable impacts on pedestrian movement and on the forces with which they encounter contact. The mechanisms of physical contact among people in highly dense areas should be investigated to measure the pedestrian crowding forces quantitatively. All of these are necessary to infer the situations of stampedes, bottlenecks, and impulse transfer in dense populations [15]. Various studies use experimental settings and modeling techniques to investigate these phenomena. Those investigations provide essential information on the behavior of crowds, which has played a role in the development of models of prediction and safety protocols.

Quantifying the forces exerted by pedestrians during contact offers distinct advantages across several methodological approaches. Pressure sensors, commonly employed in experimental settings, are used to measure forces acting on participants' bodies, particularly in crowding situations and competitive evacuations [14,16]. These sensors enable the collection of complex force patterns and forces experienced by people during peak forces. High-density areas are continuously monitored using advanced motion capture devices [17] equipped with inertial measurement units (IMUs) to monitor and record people's movements and interactions accurately. These modern technologies produce precise 3D data on segment motion and allow researchers to estimate ground reaction forces from these measurements by tracking displacements [11,18].

Numerical simulation models also serve as valuable tools for measuring contact forces. Among these, force-based models, such as the social force model and its variants, simulate pedestrian interactions arising from both physical and non-physical factors [19]. These models have been validated through comparisons with experimental data, demonstrating their ability to replicate observable phenomena such as lane formation and collision avoidance [[20], [21], [22]]. Applying computer-based simulations based on pedestrian models is a very effective way of studying such collective movement. These models are of low cost, enabling research to test scenarios that are, in almost all cases, impracticable under live experiments. Modeling escape behaviors [23], generates a shockwave that propagates through a crowd [24], or simulates the influence of the forces of crowding [25]. These models invoke the motion laws to predict the activity of individuals as a function of their preferred speed and the forces surrounding neighbors apply to them [21,22]. Validation is also conducted with the corresponding experimental data so that they reproduce precisely the crowd's behavior in the real world.

Mathematical models employing polynomial and regression analyses have been developed to describe the relationship between contact forces, velocity, and area occupancy rates. These models allow for a quantitative assessment of contact forces by correlating them with easily measurable variables such as pedestrian velocity and density [26,27]. Some studies also researched force propagation in the event of stationary crowds to a specific external perturbation like a push. The experiment design depends on applying pressure sensors and data from a motion capture system, which will provide velocity together with distance to ascertain impulse propagation [18,20]. Chraibi et al. aim to determine the effect of starting separation and crowd density on force transmission.

Research has shown that barriers can improve evacuation efficiency by reducing queuing times at exits, but only under competitive conditions. In contrast, under cooperative conditions, barriers tend to decrease flow speed [16]. How force is distributed throughout a crowd having different postures is an ongoing study in pedestrian dynamics. Controlled external forces, such as pushes, have presented a distance-dependent spreading rate of crowd density and initial distances between individuals. Higher densities mean that damping is more visible, which states the structure of the crowd and separation in building up safety more wisely. The models need continuous renovation and fine-tuning in their application to any practical situation. Preparedness influences speed and distance only negligibly impulses pass through crowds [27]. Factors defining how force distributes have a much more significant effect on that boundary, like crowd density and beginning spacing distribution parameters [28]. Measurement and analysis of forces exerted by pedestrians could produce accurate and reliable models of crowd behaviors [21].

The study of pedestrian contacting forces is getting more social and academic attention, because of the rising number and severity of crowd-related incidents [29]. Past research has tried to address the issue by developing basic observational methods and experimental and theoretical models, which didn't clearly explain how crowds behave. However, understanding these interactions has become a crucial research issue for academia and industry as cities and populations grow. Accurately measuring and modeling how people move and push against each other in crowds can help to take advanced precautions, improve the design of public areas, and prevent emergency crowd accidents [30]. At the strategic and tactical levels, advanced technology has expanded researchers' ability to study and understand complex crowd behavior in various situations. These technologies make it easier to measure pedestrian contacting forces accurately and to predict pedestrian dynamics, ensuring safer and more efficient management of large populations.

Despite these advances, early research on pedestrian contact often faces significant challenges in capturing the complexity of crowd behaviors. These challenges stem primarily from the limitations of observational methods and the inability of early experimental and theoretical models to explain the dynamic and multifaceted nature of human interaction in dense populations. For instance, many of the initial models failed to adequately account for the influence of cultural factors on the variability of individual behavior or environmental factors, which are critical to understanding population dynamics. In addition, much of the data used in earlier studies was either incomplete or prone to inaccuracy due to the lack of sophisticated data acquisition approaches. However, the integration of advanced technologies, such as high-resolution motion capture systems and real-time pressure sensors, provides researchers with tools to overcome these limitations. These advances have allowed for more accurate data acquisition and the development of models that more accurately simulate real-world crowd scenarios.

The study of pedestrian contact forces continues to offer valuable insights into crowd behaviors, providing ways to reduce the risks associated with large gatherings. While most previous studies have two shortcomings: (1) Lack of sufficient sensor-based body measurements: although most studies have concentrated on measuring forces in the back, hands, and other body parts, the chest has rarely been explicitly mentioned as a focus, except in the study [26]. (2) The lack of contact force measurement on slop roads: Most experiments have been conducted in flat areas, indoor corridors, staircases, or competitive evacuation drills. Almost no consideration has been given to the force measurement on a sloped road or a sloped micro-road network.

The preliminary contributions of this article are as follows: (1) We conducted an in-depth survey of various forces acting on pedestrians, including normal external forces, self-driven forces, abnormal external forces, and motion constraint forces from obstacles, providing a comprehensive understanding of pedestrian dynamics in mass-gathering environments. (2) Pedestrian crowding postures were reviewed, emphasizing the pedestrian crowds' impact on force distribution and dynamics and highlighting the importance of including chest and other less-studied body parts in future force measurements. (3) Advanced measurement technologies, such as pressure measurement sensors, motion capture systems, and numerical simulation methods, were systematically evaluated, offering insights into their capabilities and limitations in capturing pedestrian interactions. (4) A novel sensor-based investigation direction was proposed to fill the research gap in studying pedestrian forces on sloped micro-road networks, with suggestions for improving data transmission through wireless technologies like Wi-Fi. (5) Future research directions were outlined, including planned experiments in highly crowded environments, such as Shanghai Hongqiao Railway Station, to validate and refine the proposed methodologies, ultimately contributing to safer crowd management strategies.

The remainder of this paper is organized into the following sections. Section 2 analyzes the combination of forces acting on pedestrians, including normal external forces, self-driven forces, abnormal external forces, and motion constraint forces caused by obstructions. Section 3. explores the detailed data of crowding posture and further tests these differences in postures of the pedestrians on the dynamics of crowd flow and pedestrian safety. Section 4 describes the applied techniques used to measure the contacting forces, including the pressure measurement sensors, motion capture systems, and numerical simulation methods. Section 5 offers an overview of further studies that have the potential to expand the research on pedestrian dynamics and safety analysis. Finally, Section 6 concludes with what has been investigated and which directions should be considered in the future.

## 2 Forces in a moving pedestrian flow

### 2.1 Evolution of pedestrian force research

#### 2.1.1 Historical evolution of pedestrian force research

The study of pedestrian forces began with simple observations focused on understanding crowd behavior [31,32]. Early researchers looked at patterns like bottlenecks near exits and crowded spots, providing useful insights for designing public spaces and managing how people flow through them. As city populations grew during the mid-20th century, it became clear that more precise models were needed to understand the forces at play in crowded environments [33]. Computers made it possible to create basic computational models, but these early versions had limitations [34]. They often treated crowds as uniform groups, without considering how individual people might behave differently. Larger groups walk slower, while individuals walk faster. Studies show that as group size increases, walking speed decreases, particularly at signalized intersections and on stairs [10,35]. A significant breakthrough occurred in the 1990s with the development of the social force model [32,36]. This model used attractive and repulsive forces to represent how people interact, offering a deeper understanding of crowd dynamics. It took into account how people maintain personal space and avoid obstacles.

In recent years, technological advancements have greatly improved research on pedestrian forces. High-resolution pressure sensors now provide accurate measurements of the forces people exert [30]. At the same time, motion capture systems track movements in three dimensions, giving detailed insights into how these forces act on the body [37,38]. Additionally, There are several experimental studies that focus on pedestrian flow analysis at different levels of pedestrian densities [39,40]. Among data collection techniques, video and image data collection are the most popular. Video analysis tools have become essential for observing crowd behavior in real-time, especially during emergencies and high-density events, revealing patterns that influence how forces are generated and crowds behave [41].

#### 2.1.2 Current research hotspots

This growing interest, which started in the last decades, might be explained by the multitude of potential applications that could use the results of this research field, how pedestrian crowds behave during emergencies, optimizing urban planning, studying cultural and behavioral influences, and using advanced technologies like AI and machine learning [42,43]. A key area of interest is how people react in emergencies, such as fires, terrorist attacks, or natural disasters, where panic and sudden crowding can create dangerous levels of force [44]. Researchers are developing models to predict how crowds respond under stress and design safer evacuation procedures. These models consider psychological factors like fear and urgency, which heavily influence how people behave and generate force in emergencies [45,46]. Researchers are working on optimizing the layout of busy areas like subway stations, stadiums, and shopping malls by looking at where to place barriers, entrances, and exits and how architectural features impact crowd behavior. The goal is to ensure smooth movement, reduce congestion, and lower the risk of accidents. For these reasons, in the last decades, people detection and tracking has become an important research area in computer vision.

Research also explores how cultural and behavioral differences affect how crowds move and create forces. Cultural differences in social conventions, personal space preferences, and group dynamics can greatly impact crowd dynamics [47]. Understanding these differences is vital for developing models that can be applied globally, ensuring safety measures are effective in various settings. Individual characteristics, such as age, gender, and physical ability, also affect how people move and apply force [48]. These factors are being studied to create more inclusive and accurate models. AI and machine learning use in pedestrian force research is a growing trend, offering new ways to analyze large amounts of data from sensors and simulations. AI-driven models can spot patterns, make real-time predictions, and improve accuracy by learning from past data [49,50]. These capabilities are invaluable for managing large events and public spaces, allowing for proactive safety measures. Looking ahead, developing comprehensive models incorporating physical, social, and technological factors is a key direction for pedestrian force research. Using virtual reality (VR) and augmented reality (AR) to simulate how people interact offers new ways to understand how forces affect behavior in realistic yet controlled settings. As technology continues to evolve, the ability to study pedestrian forces more accurately and in greater detail will significantly enhance researcher's understanding of crowd dynamics, leading to safer and more efficient public spaces. Pedestrian forces are considered important factors in the pedestrian contacting forces to find and develop an accurate representation of crowd dynamics [42]. Table 1 highlights main achievements from the various works on the distribution of contact forces in crowded spaces.

**Table 1:** Methodologies and key findings in pedestrian contact force studies.

Study	Methodology	Key Findings	Reference
Interaction force analysis	Controlled impulse experiments	Standing closer together increases force magnitude and damping effects.	[18]
Systematic Experimental Investigation	Laboratory experiments with varied stances	Wider stances improve stability and reduce lateral force impacts.	[16]
Propagation of Controlled Frontward Impulses	Motion capture analysis	Sideways orientation helps distribute forces along the body's length.	[18]
Pedestrian Dynamics in Real and Simulated World	Impulse propagation experiments	Preparedness does not significantly affect impulse propagation speed.	[27]
Experimental Study on Pedestrian Contact Force	Experiments with pressure sensors	Higher competitiveness leads to increased contact forces and kinetic stress.	[51]
Contact Forces and Dynamics	Pressure sensors and simulation	Forces are concentrated in the upper body regions and increase with space occupancy rates.	[14]
Pedestrian Collective Motion in Competitive Evacuations	Evacuation drills with trajectory analysis	Higher competitiveness results in sudden collective movements and slower velocities.	[11]
Modeling of Pedestrian Motion	Simulation with visco-elastic contact forces	The model successfully simulates lane formation, obstacle avoidance, and panic behavior.	[22]

### 2.2 Normal forces on pedestrians

#### 2.2.1 Pedestrian contact forces

Pedestrian contact forces are measured and quantified dynamics to understand human behavior in a dense setting. Forces generated by pedestrians during interaction with the physical context set the requirements of infrastructure design, safety criteria, and crowd management strategies [52]. Mathematically, pedestrian contact forces can be defined using Newton's second law, which states that the force $\left(F_{\text{ped}}\right)$ The force exerted by a pedestrian is directly proportional to their mass (m) and acceleration $\left(a\right):F_{ped}=m\cdot a\text{.}$ Simulations provide an approximation of the contact force. $\left(F_{contact}\right)$ among pedestrians based on their proximity $\left(d\right):F_{contact}=k\cdot d$ where k is a coefficient derived experimentally from biomechanical models. The trends of muscle activation and joint mechanics can be investigated to understand how these factors deeply derive the contact forces. Some of the models could even have equations for muscle forces and joint torques, along with the current ground reaction force at that instant, to get a more accurate understanding of the interaction between the pedestrian and the ground.

#### 2.2.2 Obstacle forces

In very high-density areas, pedestrians will come into contact with most of their other constraints or obstacles, such as walls, barriers, and sidewalks. Physical barriers are recognized as the dominant parameter for analyzing the flow and safety of pedestrians when analyzing the consequences of interaction induced by the forces of obstacle interaction. These forces arise from the interaction between pedestrians and the obstacles formed by their movement. The repulsive force between a pedestrian and an obstruction is defined by one of the primary equations of the social force model:

(1) $$F_{ob}=k\cdot e^{-\frac{d}{r}}$$

where k represent the interaction strength, d is the distance to the obstacle, and r is the effective interaction range [32]. Furthermore, it also analyzes the density as well as velocities of pedestrians to measure forces that appear in high-density cases and in light of these the formula is developed $P=\rho .v^{2}$, where ρ is the density and v the velocity of the pedestrian flow [53]. To accurately find out the effect of different influences, experiments frequently use:

(2) $$F_{total}=F_{ob}+\mu .N$$

where μ represents the contact ratio between the pedestrian and the obstacle, and N is the normal force the pedestrian exerts [19].

### 2.3 Pedestrian self-driven forces

Pedestrians in a crowd avoid obstacles in a way that gives rise to some sort of force. The pedestrian dynamics are guided by a set of forces in which actions are deduced so that the pedestrians do not bump into each other, and it is a smooth flow situation in which each one of them is guaranteed safety. From the social force model, pedestrian avoidance behavior is modeled and parametrized from the point of view where every pedestrian generates a repulsive force to maintain personal space. The avoidance force $F_{av}$ between two pedestrians can be expressed as:

(3) $$F_{av}=A\cdot e^{-\frac{d-r}{B}}\cdot \hat{n}$$

where A and B are empirical constants, d is the distance between the centers of two pedestrians, r is the sum of their radii and n is the unit vector pointing from one pedestrian to the other [32]. The mathematical model can represent the required change in direction to avoid collisions when considering pedestrians' speed and direction as:

(4) $$\Delta \vec{v}=-k\cdot \left(\vec{v}-{\vec{v}}_{opt}\right)$$

where k is a constant reflecting the rate of change in velocity, $\vec{v}$ is the current velocity and ${\vec{v}}_{opt}$ is the optimal velocity to avoid contact [54].

Further expanding on this model, an angular dependency to account for the pedestrians' field of vision, refining the repulsive force as:

(5) $$F_{av}\left(\theta \right)=A\cdot e^{-\frac{d-r}{B}}\cdot e^{-}\frac{|\theta |}{\theta_{0}}$$

where θ is the angle of interaction relative to the forward direction of motion, and $\theta_{0}$ represents the width of the effective interaction field [55].

Crowd contact dynamics are equally important with the forces of pedestrian self-driven, to describe individual movement within a crowd, especially in high-density crowds. Recent literature has been increasingly focused on incorporating these contact dynamics into pedestrian models. For instance, Gao Y. et al. (2017) presented an improved social force model accounting for body compression and contact force effects to build a more realistic modeling of pedestrian behavior for high-density situations [56]. To predict pedestrian dynamics in a corridor where a camera or laser cannot meet monitoring requirements, a crowd hybrid model combines the benefits of microscopic pedestrian movement models, which consider individual interactions in detail and macroscopic pedestrian movement models, which require less computation [57]. The study by Li, C. et al. (2023) improved the Aw-Rascle fluid dynamics model by incorporating a pressure coefficient and disturbance intensity to enhance the pressure term. It also developed an internal disturbance propagation model (DPM) for fall behavior.

Damping motion theory was employed to suppress disturbance propagation caused by falls, and the disturbance elimination mechanism was discussed. The model's validity was confirmed through field experiments and numerical simulations [58]. Zhao, R. et al. (2023) proposed a novel dynamic centroid model (DCM) of a human body and rebuilding pedestrian joint sub-segments from human skeleton key nodes obtained in camera images and showed that the model was capable of detecting abnormal behaviors [59]. Zhuang X. et al. (2018) implemented a model in which the effect of physical contact can be integrated into the pedestrian decision-making process, showing how these factors might lead to emergent behaviors like bottlenecks and shock waves [60]. Meanwhile, Huang S. et al. (2024) applied optimization through neural network approaches in pedestrian dynamics studies for the parameter estimation of contact forces within these models to arrive at better predictions of crowd movements under a variety of conditions. These developments show the relevance of accounting for crowding contact in the analysis of self-driven forces, bringing more understanding to pedestrian dynamics and enhancing the effect of crowd management strategies in real-world situations [61].

### 2.4 Abnormal force to pedestrian

No matter whether pedestrians are transiting from one current location to another or are stable standing in the same place, abnormal external forces can sometimes lead to crowd accidents. These abnormal external forces can be physical contact with another person or an object, sometimes pushed by a vehicle or an object, an accident like a sudden fall down, environmental factors such as heavy winds, and purposefully abnormal acts such as hitting by another person such as terrorism attacks, assault or violence. One basic model for the force exerted by one pedestrian to another is derived from modifications to the classical social force model proposed by Ref. [32]. This model describes the exerted force $F_{ex}$ as a function of both the compression (R−d) and the sliding friction influenced by relative tangential velocities Δv, formulated as:

(6) $$F_{ex}=k\cdot \left(R-d\right)+\kappa \cdot {\left(R-d\right)}^{+}\cdot \Delta v\cdot \hat{t}$$

where k and κ denote the elastic and sliding coefficients, respectively, and $\hat{t}$ denotes the tangential unit vector [62]. Additionally, the pressure P Environmental structures experience substantial stress, particularly in densely populated areas, which can be measured by:

(7) $$P=\frac{F_{ex}}{A}$$

where A represents the contact area, influenced by the crowd's density and physical arrangement [63]. To better accommodate the changing levels of crowd density, an adjustment factor can be integrated into the abnormal exerted force model, thus enhancing its ability to anticipate outcomes in various crowd densities accurately:

(8) $$F_{ex,d}=k_{d}\cdot \exp\left(-\frac{d-R}{R_{0}}\right)\cdot \left(1+{\frac{\rho }{\rho }}_{0}\right)$$

This modification introduces $k_{d}$, $R_{0}$ and $\rho_{0}$ Employing parameters that modify the force according to empirical measurements of crowd density establishes a more flexible framework for simulations [36].

### 2.5 Motion constraint forces from obstacles

Motion constraint forces are exerted on pedestrians because of constraints or barriers that reduce their movement. This obstacle could be static, like walls, other fixed structures, moving vehicles, or pedestrians. This interaction could play a preeminent role in movement patterns and flow efficiency. An increased understanding of these forces, therefore, is critical to maintaining the stability of the crowd or, rather, understanding the crowd dynamics. The most straightforward equation available for describing the interaction between pedestrians and stationary environmental limitations is formulated as follows:

(9) $$F_{mc}=k_{c}\cdot e^{-\frac{d}{\delta }}\cdot \hat{n}$$

where $F_{mc}$ Represents the motion constrain force, $k_{c}$ Represents the stiffness constant, which quantifies the repulsive effect of the boundary, d is the minimum distance from the pedestrian to the obstacle, δ indicates the effective range over which the force is felt and $k_{c}$ is the unit vector normal to the obstacle pointing towards the pedestrian [32]. To be able to accommodate different pedestrian velocities and the density of local crowds, the equation can be enhanced to incorporate these dynamic factors:

(10) $$F_{mc,v,\rho }=k_{c}\cdot e^{-\frac{d}{\delta }}\cdot \left(1+\alpha \cdot v+\beta \cdot \rho \right)\cdot \hat{n}$$

where α and β are coefficients that modulate the influence of velocity v and density ρ, Respectively, providing a more responsive interaction model that reflects real-world conditions more accurately [36]. In addition, this can be used to introduce an angular dependence into the force equation for the force being used to describe motion systems if a directivity exists in the system and, in turn, the troops are restricted to be dependent on an angle whereby a pedestrian approaches a boundary:

(11) $$F_{mc,dir}=k_{c}\cdot e^{-\frac{d}{\delta }}\cdot \cos\;\left(\theta \right)\cdot \hat{n}$$

where θ is the angle between the pedestrian's movement direction and the normal to the obstacle, affecting how the experience of force depends on the direction of the approach [64].

Although representing pedestrian contact forces through model formulas is important, it is equally crucial to explore the parameterization of these models for a better understanding of the underlying dynamics. For example, in the equation $F_{contact}=k\cdot d$ where k is a coefficient derived from biomechanical data, attention should be paid to how this parameter varies with factors like pedestrian density, body mass distribution, and environmental conditions. Similarly, in obstacle force models, parameters such as interaction strength k and effective range r should be examined in detail, considering their dependence on crowd movement speed, obstacle material, and proximity. The variability and sensitivity of these parameters above can significantly impact the predictive accuracy of the models. Therefore, by incorporating a more thorough discussion of these parameter issues, the models can be further refined to capture the complexities of pedestrian dynamics better, moving beyond simple force representation to foster a more nuanced understanding of interactions.

## 3 Pedestrian crowding posture

This section introduces the different crowd-standing positions, real-world experiments from recent studies, and the researcher's valuable findings and how they affect the crowding forces. In a crowded condition, where and how pedestrians stand is vital in estimating the strength of the force people experience. The posture, equilibrium, and reaction of pedestrians have a preliminary impact on the transmission and absorption of forces within a crowd [65]. Pedestrian crowding posture refers to the way people stand and move in crowded spaces, especially when they encounter sudden disturbances. These postures can cause local turbulence, interrupt the smooth flow of pedestrians' movement, and create differences in speed and density within the crowd. Identifying these unusual postures using techniques like motion-capturing systems and computer vision can help detect potential dangers early, which can be crucial in preventing incidents like stampedes [66]. An early study by D. Helbing et al. points out that people tend to adopt postures that reduce the risk of physical contact and preserve their personal space in crowded environments. The crowd's movement shapes this behavior, as individuals adjust their position and stance to avoid bumping into others and keep their balance [42].

**Fig. 1:**
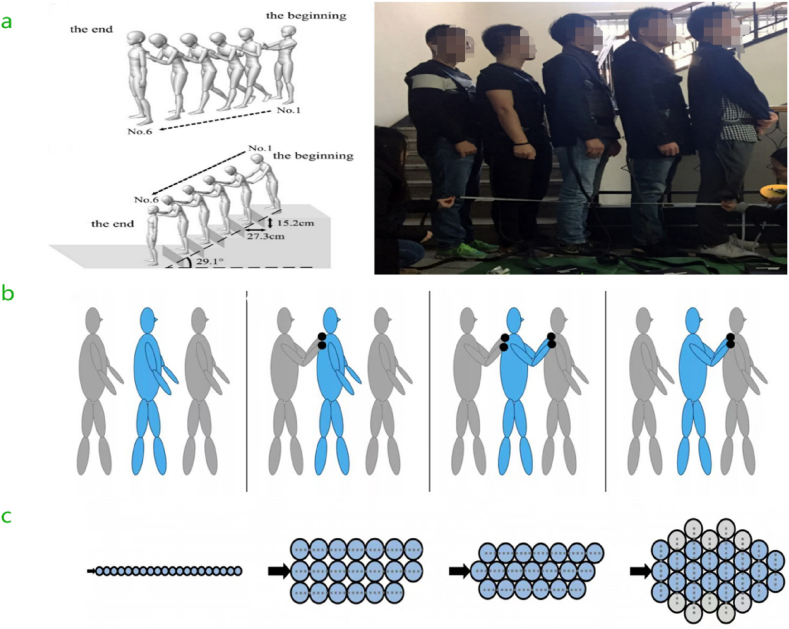
Typical crowd-standing postures. a) The flat surface and the staircase condition standing postures with only difference in the first image allowed hand protection, and the second image did not [13], note: To protect their privacy, the faces of the image have been blurred. b) Pedestrians without touching each other, the first touching the second, the first and second touching the third, and the second touching the third [67]. c) Participants stood in a queue of 20 people, three to five rows side by side, and relocated to the front, with one person having the shoulders of two in front of them [18].

The pedestrian postures, including their stance width, body orientation, and balance, impact the size and distribution of contact forces in overcrowded environments. Conducting controlled experiments to investigate how varied postures and spatial arrangements impact the forces pedestrians experience on contact. The performers included six males between 20 and 30 years of age tested under both test scenarios on level ground and inside a staircase with an inclination angle of 29.1° [13]. In the experiments, participants were positioned linearly at different spacings, ranging from 0 cm to 30 cm, to simulate real-world crowding scenarios (see Fig. 1-a) where accidents are likely to occur. Two experimental conditions were created: one without hand support and another where hands were used to brace against collisions. The introduction of a slope to the Stairs experiments brought the forces of gravity into play, thus raising the level of complexity. The results showed considerable variations in collision force pulses depending on the posture of the individuals in crowded situations, with variations in both shape and magnitude. Force pulses for distances less than 30 cm generally displayed a bimodal pattern resulting from rapidly repeated initial contact and stability occurrences. Closer distances were associated with larger collision forces, indicating more severe impacts. Insufficient hand protection resulted in increased contact forces transferred through the upper body, significantly influencing stability and balance. Numerical data demonstrated separate stages of the collision: touch, squeeze, bounce, and separation.

Without hand support, collision forces reached larger peaks. In contrast, the ability to use hands lowered impact pressures on the upper body by absorbing and spreading energy, resulting in lower collision force peaks. On the staircase, the sloped position caused a downward pull caused by gravity, which changed how collision forces worked and led to different features compared to situations on a flat surface. Participants encountered a blend of propulsive and gravitational forces, resulting in an apparent sequence of collision force pulses. The numerical findings clearly showed that the forces resulting from collisions were higher when pedestrians were in closer proximity. Additionally, using hands played a crucial role in reducing these forces. An angled posture on the stairs adds complexity to the analysis of force dynamics, highlighting the importance of including horizontal and vertical crowding postures in pedestrian dynamics models. Research shows that the starting distance between individuals and their alignment can affect how forces spread in a crowd [18]. For example, closer proximity between individuals leads to stronger forces and greater reductions in the effects of external impulses.

Experiments involving controlled pushes (see Fig. 1-b) have shown that the speed and distance of impulse propagation are significantly influenced by the participants' standing postures. When participants were arranged in staggered formations, with each individual positioned between the shoulders of two people in front (see Fig. 1-c), the force was distributed more evenly. This arrangement resulted in a reduction in the maximum forces experienced by any single person [21]. A study by Mira Beermann and Anna Sieben (2023) examined the effects of pedestrian crowding and reduced walking speed due to increased density on stress levels, using electrodermal activity (EDA) sensors. In this experiment, participants walked in a confined space under varying levels of crowd density, which led to decreased walking speeds and increased physiological arousal, indicating higher stress levels. The findings demonstrate that stress levels rise as density increases and personal space decreases, suggesting that both the number of individuals in close proximity and restrictions on body movement significantly influence individual stress in crowded situations [68]. Additionally, a study by H. Kao et al. (2022) observed pedestrian behavior on a busy street near the University of California, Berkeley. The study measured pedestrian interactions and behavior in high-density settings. Post-crowding analysis revealed that pedestrians often adopted characteristic formations that facilitated movement in dense crowds, such as bunching up and orienting their bodies to avoid collisions with others [65]. This configuration mimics real-world crowd circumstances where individuals frequently assume non-uniform positions, resulting in intricate force distributions.

Adopting a broader posture decreases the probability of falling or being forcefully moved when subjected to external pressure [16]. Individuals who encounter the direction of force propagation are more vulnerable to experiencing direct impacts. In contrast, those orientated sideways can more effectively distribute the force along their body length [18]. The capacity to sustain equilibrium in the face of external influences is paramount. Individuals who are well-prepared and able to anticipate a force might position themselves and assume a stance that reduces the impact. Nevertheless, studies have indicated that readiness does not have a substantial impact on the rate at which impulses spread. This implies that other parameters, such as density and starting spacing, have a more critical influence [27].

## 4 Model of contacting force measurement

In recent years, many researchers have used different sensors, models and equipment to measure pedestrian contacting forces. Such as the TekScan 5400N and Tactilus®, strategically placed on key body parts like the back, shoulders, elbows, and feet to capture contact forces in crowded situations. Their experimental setup was carefully designed, ensuring the sensors were highly sensitive and accurate, capable of registering forces up to 650 N. To minimize measurement errors, techniques like noise reduction and sensor drift compensation were employed. Data was meticulously computed and stored, allowing for real-time collection and detailed post-processing, which revealed important insights, such as the exponential relationship between crowd density and force magnitude. Their findings significantly contribute to pedestrian crowd stability and dynamics (Table 2). shows some recent studies and their findings.

**Table 2:** Recent study on pedestrian contacting force measurement and findings results.

Study	Measurement Type	Sensor Used	Measured Body Parts	Finding results
[25]	Crowding Forces	Wearable Pressure Sensors	Back, Shoulders, Upper Limbs	Pressure on the back is greater than on the shoulders, influenced by crowd density. The highest force measured 90.2 N.
[69]	Contact Crowding Force	Pressure Sensors	Back, Arms	The highest crowding force reached 90 N. There is a Clear exponential relationship between force and pedestrian density.
[21]	Individual Response in Dense Crowds	Xsensor, TekScan5400N	Back, Head, Elbow	The highest force is 90.2 N, and the wall impact is greater than the punching bag.
[26]	Contact Forces in Crowding Situations	Flexible Fabric Pressure Sensor	Breast, Shoulders, Upper Back, Intrascapular Regions	Contact force increased linearly with SOR from 77.8 N to 506.3 N. Upper body force distribution was observed.
[70]	Impulse and Release Distance	Tactilus® Pressure Sensors	Back, Head, Shoulder, Hand, Knee, Foot	Substantial rise in release distance with increasing impulse.
[13]	Collision Forces	Tactilus® Pressure Sensors	Back, Hands	Greater centroid distances are associated with higher collision forces.
[14]	Contact Pressures in Evacuation	TekScan5400N	Back, Head, Elbow, Foot	Higher pressures at doorjambs a significant increase in high-competitiveness situations.

### 4.1 Pressure measurement sensors

**Fig. 2:**
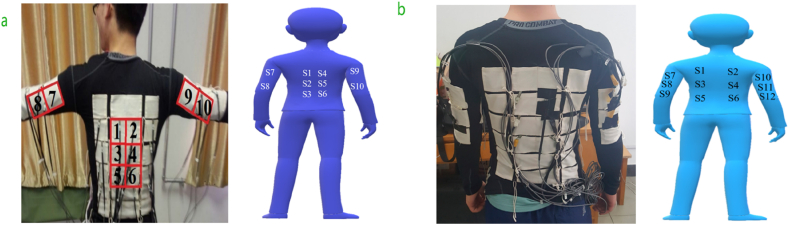
wearable sensors and their attachment position on the different human body parts. a) Show a person attaching six sensors on the back part of his body, with each shoulder attaching two, and b) attaching six on the back and both shoulders attaching three [25,69].

Measuring pedestrian contacting force is challenging because of the intricate interplay of pedestrians' physical and psychological behaviors. However, advancements in sensor technologies have facilitated the development of wearable pressure-measuring sensors and fabric pressure-measurement sensors, which offer promising avenues for accurately quantifying pedestrian-pedestrian contacting force or pedestrian obstacle contacting force. Using Pressure Measurement Sensors, Song et al. (2019) [25] measured the forces exerted by overcrowding in a tube carriage in Beijing during the busiest hours. An experimenter was outfitted with specialized clothing, including pressure sensors. Then, the experimenter went back and forth on different tube lines during the busiest hours of weekdays. As shown in Fig. 2-a, the sensors were appropriately positioned on areas of the body prone to bending, specifically the back and both upper limbs. The sensors used in the experiments were sensitive enough to respond to even small changes in pressure. The most challenging part of data processing is noise elimination and sensor drift compensation, which is highly important given that the crowd is a constantly changing and moving system.

**Fig. 3:**
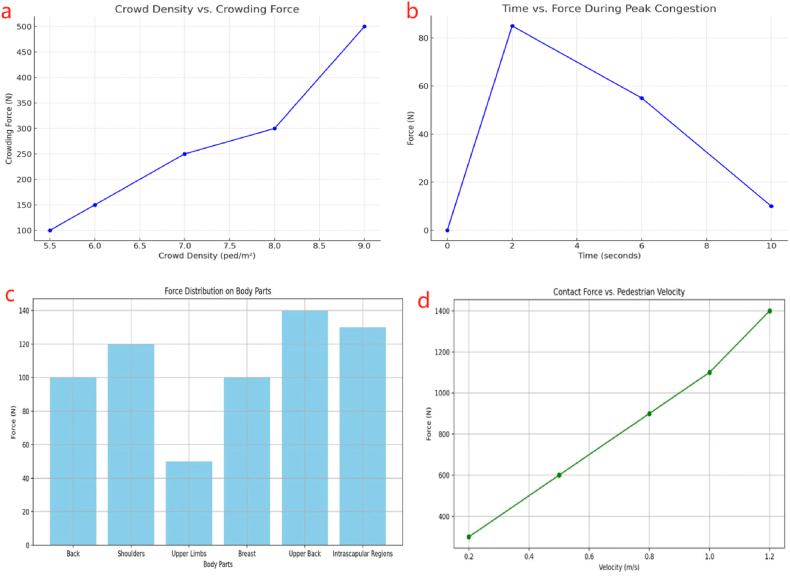
Typical experiment results in data charts from different [25,51,69,70] studies. Which include a) crowd density vs crowding force. b) time vs force during peak congestion. c) force distribution on body parts. d) contact force vs. pedestrian velocity.

The findings showed that the highest recorded crowding force reached 90 N at the most densely populated location, indicating a clear exponential relationship between crowding force and pedestrian density. The greatest force values were seen near the narrowest point, reaching a value of 90.2 N at a time of 2.4 s during the period of heaviest congestion [25]. The forces substantially decreased after 6.8 s, suggesting a decline in pedestrian encounters as crowding cleared. As shown in Fig. 3-b, the experimental results agreed with the simulated outcomes, with an acceptable error range of 1.3 %. The data acquired included crowd density and its related crowding forces, which were documented and examined to comprehend the correlation between crowd density and the highest level of crowding force. The pressure exerted on the back was consistently greater than on the shoulders, and the crowd density notably influenced this difference (Fig. 3-a). shows the measured contact crowding force among pedestrians in densely populated situations [69]. During the experiment (see Fig. 2-b), twelve pressure sensors were attached to the participant's clothes to measure the extent of the forces generated in high-density situations developed by Sensor Products Inc., USA. The sensors were placed in grids with six on the back and three on each arm. The size of each square sensor was 0.044 m per side. The sensitivity of the pressure sensor was 0.001 KPa, and the frequency was 50 Hz. In this experiment, designed to model high-density or bottleneck conditions, the maximum measured crowding force was 90.7 N at 2.4 s for 0.1 s, mostly on the participant's right arm nearest the bottleneck. The relationship between crowding force and pedestrian density was exponential, stabilizing as density reached 8.2 ped/m^2^. Further data processing focused on extracting maximum force values at regular intervals and analyzing the distribution of this force across different body regions.

Yue et al. used the Xsensor LX210:50.50.05 (xsensor, 2019) [71] pressure measurement sensor. With this configuration, the researchers could investigate how these forces spread through groups of individuals. In the Latent Differential Physics (LDP) model framework, the researchers used differential physics and deep neural networks to capture and predict the implications of physical disturbances. This setup allowed for the ability to measure human responses to motion at 60 Hz, and it recorded data very precisely. Data collection for force magnitudes in the experiments was recorded in weak, medium, and strong, and the highest force magnitude recorded was at 0.623 m Mean Per Joint Position Error (MPJPE). All forces were hand-applied by the same operator on each trial to ensure that the experimental design was the same on each trial [72]. The maximum pushing force was recorded as roughly 37.36 % higher than the most potent starting push in the dataset. The measurements showed notable differences in contact forces based on the intensity of the disturbance, with higher pushing resulting in greater recorded forces.

Another experiment by (Feldmann & Adrian, 2023) [21] measured a person's response to external forces in dense crowds. They used an Xsensor, model LX210:50.50.05, and a TekScan 5400N model pressure sensor. The Xsensor, attached to the punching bag, had 2500 measuring cells spread over an area of 25.4 cm × 25.4 cm, recording pressure at 34 frames per second with a pressure range of 0.14 N/cm^2^ to 10.3 N/cm^2^. The wall-mounted TekScan sensor covered a larger area—57.8 cm × 88.4 cm, 1768 measuring cells—and provided the sensitivity required to record pressures at 90 frames per second, with a pressure range further tailored through pre-calibration using weights of 120 kg and 185 kg to improve sensitivity. The intensity of the applied pushes was varied (weak, medium, strong), and the resulting forces on the punching bag and wall were measured. The highest impulse measured was 90 N s when participants were spaced at elbow distance, which the participants perceived as the most dangerous scenario. The forces exerted on the wall are much higher than those on the punching bag. This difference is linked to the various times of contact and multiple sensitivities of the sensors to the impact. It also investigated the distance and velocity at which a push propagates through a line of five individuals. Li, Song et al. study demonstrated that the amount of force applied by the push changed according to the initial distances between persons and the existence of a wall, which directly influenced the intensity of the measured forces [51]. They Used a flexible fabric pressure sensor to measure the contact pressures between individuals in various crowd environments. The sensor, equipped with 1024 sensing points evenly spread over a surface spanning 46.5 cm × 46.5 cm, collected force data in real-time at a maximum frequency of 50 Hz (Tactilus®) [73]. The measurement has an accuracy of ±10 %, a precision of ±2 %, a hysteresis of ±5 %, and a non-linearity of ±1.5 %. Findings indicated that pedestrians experienced no discomfort when subjected to contact forces, provided that the Space Occupancy Ratio (SOR) remained under 0.8. With an increase in SOR from 0.57 to 0.87, the contact force exhibited a significant linear correlation, rising from 77.8 N to 506.3 N. The contact force reached 300 N under conditions of medium crowding and increased to 500 N in cases of extreme overcrowding. Analysis revealed that the force distribution was primarily concentrated in the upper body, including the breast, shoulders, upper back, and interscapular regions see Fig. 3-c. The contact forces in both static and dynamic conditions. It discovered that dynamic contact forces escalated with pedestrian velocity, ranging from 300 N to 1400 N, as velocity climbed from 0.2 to 1.2 m/s (see Fig. 3-d).

**Fig. 4:**
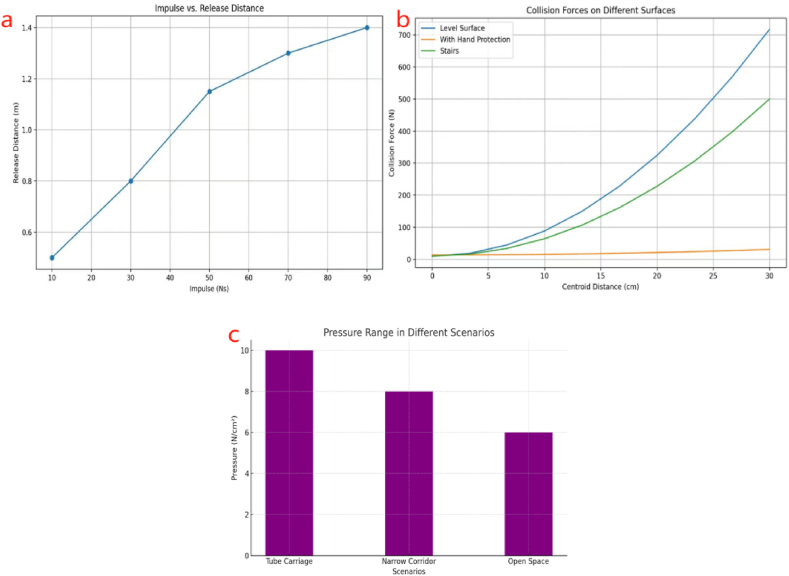
Shows the a) impulse vs release distance, b) collision forces on different surfaces, and c) pressure range in different scenarios data charts from Refs. [13,14,21] their study results.

Using the Tactilus® pressure sensor, another study on measuring the contact forces experienced by pedestrians under sudden contact forces by Xudong Li et al. (2021) showed a substantial rise in the required release distance when the impulse escalated from 10 Ns to 90 Ns (See in Fig. 4-a). The experimental setup presented scenarios where a pedestrian was subjected to a sudden push, simulating the dynamics in real-life crowds. In this setup, Tactilus® software was used to visualize and export real-time data provided by pressure sensors on force distribution for further analysis. Results showed that the distance of impulse release increased linearly with the applied impulse, with a proportional coefficient ranging between 0.01991 and 0.02847 for different participants. The peak force recorded was above 200 N, indicating that sudden contact has significant consequences in high-density crowd situations. During data processing, noise was filtered out, and the pressure data was synchronized with motion capture data to accurately track movement and force distribution across the participants' bodies. Transmission efficiency positively correlated with proximity, but correlation negatively correlated with distance. A direct correlation was seen between the applied impulse, the distance of release, and the maximal momentum. For example, a 50 N-second impulse required a release distance of 1.15 m, creating a maximum momentum of 115-kg meters per second. The results emphasize the dangers of increased urges in crowded environments. The measurement errors were not mentioned.

Nevertheless, the experimental setup ensured a high level of accuracy and reliability. Conducting controlled tests to quantify the forces involved in pedestrian collisions using Tactilus® pressure sensors [70]. In experiments by C.Wang and W.Weng the Tactilus® Attachable fabric sensors were used on human subjects, measuring 32 cm × 32 cm with 1024 sensing points. They were attached to the back for real-time pressure distribution during collisions between them under controlled experiments simulating a queue situation to measure pedestrian-to-pedestrian collision forces. The sensors were rated at 50 Hz, 0–5 PSI (0–34.475 kPa) range, and gave accuracy within ±10 %, repeatability of ±2 %, and hysteresis of ±5 %. The participants were asked to stand in a row, approximating a queue scenario on both level surfaces and stairs. The experiments were done in conditions 1 and 2 on a flat surface. The difference is only whether the hands are allowed to protect themselves actively. Condition 3 was on the staircase. Measuring collision forces from pushes behind a participant in the line, resulting in the force being transmitted into the queue. The great differences in the collision force, with the greatest forces being measured between participants at a shorter distance, for example, up to 2049.6 N in some conditions, were measured. Post-processing data for analysis of the collision dynamics has shown that the force generally increased with a decrease in the distance between the two participants and, in general, generated a higher force on the stairs than on the level surface due to the added component in gravity. The situations were conducted with a total of six male volunteers within the age range of 20–30. Participants were arranged in a linear formation and exposed to forces that imitated the cascading impact of falling dominos. Collision forces were measured at different distances ranging from 0 cm to 30 cm, both with and without using hands as a protective measure. Results indicated in (Fig. 4-b) that greater centroid distances were associated with higher collision forces, described by the fitting formula $F_{\mathrm{MAX}}=10.36814+0.78479{\left(L\times d^{2}\right)}^{2}$ in Condition 1. In Condition 2, with hand protection, forces were slightly reduced $F_{\mathrm{MAX}}=13.76856+0.01953{\left(L\times d^{2}\right)}^{2}$ In Condition 3, on stairs, forces followed $F_{\mathrm{MAX}}=10.27164+0.5438{\left(L\times d^{2}\right)}^{2}$ They analyzed the data by filtering out noise and synchronizing the pressure data to ensure accuracy [13].

Zuriguel et al. (2020) measured contact pressures during evacuation drills. The Tekscan 5400N pressure mapping sensor was positioned on the left side of the door and covered with black removable plastic. It was matched by a matching liner on the right side. The sensor comprised 1768 individual sensors arranged in a grid of 52 by 34, extending an area of 88.4 cm by 57.8 cm. Sensors were installed on the doorjamb and the nearby wall to monitor the pressure exerted as 180 soldiers exited through a narrow doorway under different competitive situations. Each unit measured 1.7 cm by 1.7 cm. The sensors were highly sensitive and could record pressure at 256 different levels. Before the experiments began, the sensors were carefully calibrated to avoid saturation and ensure precise pressure readings. The experimental setup was ready for low and high-competitiveness scenarios with and without obstacles to be placed near the exit. The pressure data showed that the highest pressures, beyond 250 units, were recorded at the doorjamb during high-competitiveness evacuations without obstacles. The peak pressures at the doorjamb decreased with an obstacle in place, especially when the obstacle was closer to the door. The pressures at the doorjamb were larger than those on the wall, especially in high competitiveness (HC) situations [14]. A wall located near the entrance impacted the distribution of pressure, producing a reduction in certain situations and an increase while in proximity to the door. Higher levels of competitiveness led to more people and congestion near the exit, with the highest density levels reaching about 7 m^2^. The results indicated significantly reduced pressure levels compared to high-pressure ones in non-competitive situations (Fig. 4-c). shows Doorjamb pressures substantially increased in (HC) situations [16].

### 4.2 Motion capture systems

**Fig. 5:**
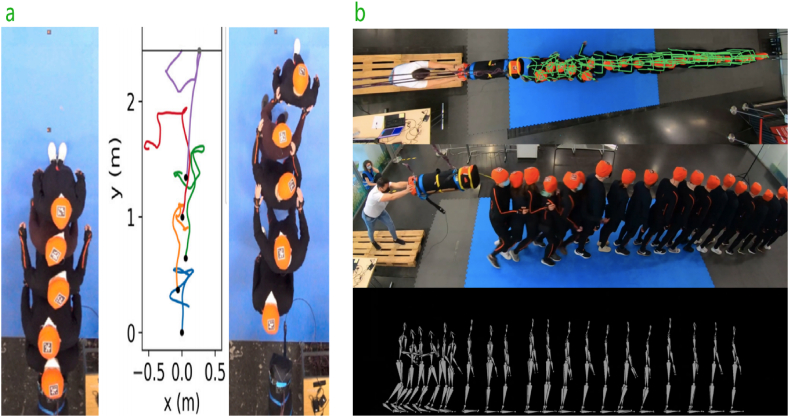
a) and b) Show the studies' methodologies to detect pedestrian motion capture in Refs. [18,21].

Motion capture technologies have become important for capturing pedestrian motion dynamics, capturing pedestrian posture, and measuring pedestrian contact forces. These systems accurately track the motions and activities of pedestrians while they are moving about using a variety of sensors and cameras. Sina Feldmann and Juliane Adrian used Xsens motion capture equipment [74] and video recording combined to measure the impulses and movements of pedestrians during tests. The primary system used was a stereo camera device called the Bumblebee XB3, with a native resolution of 1280 × 960 pixels and a horizontal field of view of 66°. The cameras were mounted overhead and aimed downward, perpendicular to the direction of pedestrian movement, to reduce occlusion and provide a clear view of every individual participating in the study. The system could capture up to 16 frames per second, ensuring even fast movements were well recorded. Controlled environments like bottlenecks and corridors were set up to monitor pedestrian flow closely. Both marked and markerless tracking techniques were used, with stereo recordings generating disparity maps that provided depth information. This allowed for accurately tracking pedestrians' movements in three-dimensional space, even in dense crowds. The system was calibrated before each experiment to ensure accuracy, with particular attention to lens distortion and camera synchronization. Robust algorithms, such as the pyramidal iterative Lucas-Kanade feature tracker, were implemented to minimize tracking errors by calculating optical flow and adjusting tracking results based on successive Gaussian pyramids of images. Data processing involved filtering out noise, correcting tracking inconsistencies, and combining trajectory data from multiple camera views using least squares methods to reduce errors. The final output provided highly accurate trajectories, with an average error of 1 cm in 2D experiments and 3 cm in 3D experiments. To collect head trajectories, participants wore orange hats with markers that were monitored using PeTrack [37,75]. Tentacle Sync E is used to sync video recordings with 3D motion data (see Fig. 5), such as acceleration and angle rate, acquired by Xsens Suites equipped with 17 inertial measurement devices. The data, including the center of mass (CoM), were analyzed to understand the propagation of impulses. Higher impulses were shown to be connected with more pedestrian movements, and thorough equipment calibration reduced mistakes. This complete setup allowed for a detailed analysis of pedestrian dynamics and the impact of external forces [18,21].

The Latent Differential Physics model (LDP) model can reliably predict motion. With some of the most significant disturbances, like those between one single object and multiple objects, but all being compared to standard methods, the model makes it easy to see that there are clear improvements when predicting complex relationships and the spread of forces. The human motion under unexpected physical perturbations was recorded using a hybrid motion capture system that combines optical and inertial technologies. The primary optical system was a Vicon setup featuring 12 infrared cameras operating at a sampling rate of 120 Hz. Twenty-two reflective markers were placed on joint locations to capture three-dimensional movement, allowing for precise tracking of full-body motion in response to external forces. Controlled perturbations were introduced using a pendulum-based device that applied varying forces to the upper body to study participant responses. Data from both systems were synchronized and processed to reconstruct full-body motion, with efforts focused on minimizing errors from marker occlusion and IMU drift. This filtering and data fusion process ensured the final motion trajectories were highly accurate, with an average positional error of less than 1 cm [72]. The advanced technique that can provide both head location and movement on a global scale simultaneously, capturing the entire motion needed for the whole of the body, is the hybrid tracking system (HTS). It combines the technology in the inertial measurement unit with the technology in the camera-based devices. This study's principal inertial measurement unit (IMU) system was the Xsens MVN Awinda, which consisted of 17 sensors strategically positioned on different body segments to capture and analyze 3D full-body motion. 3D correct measurement with an IMU-based motion capture system means that every IMU is attached to a specific segment of the body part to gather data in terms of orientation, acceleration, and angular rate, and some are even attached to account for the magnetic field intensity. The tested experiment of the IMU system connected to 25 subjects presented an error of 1.5 cm. This hybrid system can be made maximally efficient for measuring head trajectory; as such, it shows relatively high levels of motivation in the participants, resulting in the increase of velocity by 0.34 m/s and of rotation angles of the pelvis [76].

During controlled studies [67], repeated pushes were applied, and the responses were recorded as described above, using the side-view cameras and MoCap devices. Three clearly defined motion phases were identified from the measured data: impulse absorption, impulse transmission, and post-impulse stabilization. Considering several methods, from calculating the distance between points and lines in a two-dimensional plane to measuring speed acceleration forward and a projection of three-dimensional points on planes, the results showed an average of 0.67 s for the subject to attempt forward movement in response to the impulse and 2.20 s into the impulse to attain a stable position.

### 4.3 Numerical simulation methods

**Fig. 6:**
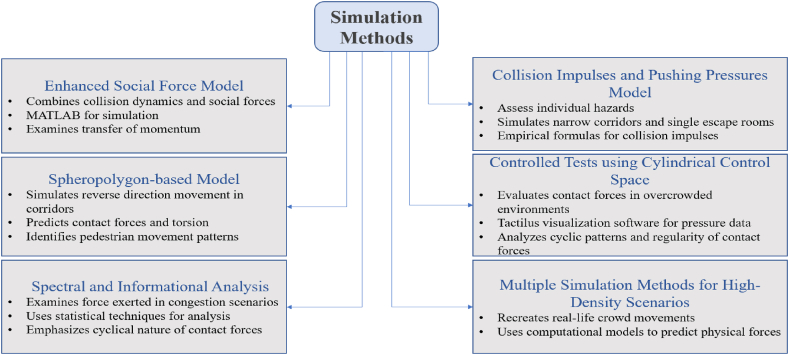
The block diagram of several Numerical Simulation Methods for measuring Pedestrian Contact Forces.

Several advanced simulation approaches have been applied to the research on pedestrian dynamics and the measurement of contact forces in the analysis and predicament of the crowd's behavior under different conditions (See Fig. 6). Some of these novel techniques have to detail the complex reactions and dynamics within crowded environments to elicit beneficial data upon testing for its safety and hazards. The numerical simulation of an enhanced Social Force Model was carried out to study pedestrian collective movement during competitive room evacuation, integrated with a kinetic stress component. The simulations are meant to reproduce pedestrian motion dynamics under different levels of competitiveness, with particular attention to crowd turbulence and kinetic stress. Calibration was based on data from controlled evacuations, focusing on variables observed during the drill—density, velocity, and kinetic stress. The internal stress from contact and pushing, which describes the kinetic stress component, plays an important role in realistically modeling sudden collective movements and turbulence in high-density scenarios.

A technique based on coarse-graining converts data for discrete pedestrians into continuous fields for density and velocity, enabling the calculation of kinetic stress and, ultimately, predicting crowd turbulence. Multiple small iterations aimed at minimizing error iteratively improve the results to match observed data closely. A study on the measurement of contact force between pedestrians applied an extended model of social force to come up with a realistic depiction of the cascading impact within lines of pedestrians. The method combined collision and social dynamics by representing contact impulse and forward-leaning pressure. All simulations were carried out in MATLAB. This model accounted for the transfer of momentum between pedestrians and further researched the contrast of the opposing forces, which can be of forward momentum and are the lunar components involved when leaning forward [11,12]. A distinct study simulated individual hazards in crowded settings, emphasizing small corridors and rooms with a single exit. The model combined two fundamental forms of physical interactions: collisions and active pushing. Interaction between individuals was modeled by combining collision impulses and pushing forces. Unlike traditional models relying solely on pushing forces or momentum conservation, this approach addressed the acceleration error by incorporating empirical formulas considering human physiological acceleration limits. The simulation model was calibrated using experimental data, including contact forces and collision impulses measured in controlled laboratory settings. These were recorded with Tekscan® pressure sensors and then analyzed to refine the model's parameters. Validation was successful, as the model realistically reproduced crowd behaviors observed in experiments, such as the human domino effect and individual crushing risk. The formulation included empirical equations for collision impulses to represent real-world movements accurately [77].

A model based on spheropolygon was employed to explain the coordinated movements of pedestrians moving in opposite directions in the corridor. This study introduces a model for simulating conservative and dissipative interactions between 2D complex-shaped rigid bodies. In this model, a pedestrian was portrayed by spheropolygons and can accurately anticipate the contact forces, ground reaction forces, and torsion pedestrians feel. The spheropolygon approach allows for the simulation of non-convex particles in all their complexity without breaking them down into simpler shapes, thereby preserving the accuracy and efficiency of the simulations. This was implemented using object-oriented code written in C++ for detailed simulation of contact interactions between particles. The key innovation lies in the Minkowski sum, which simplifies the representation of complex shapes and enables efficient calculation of contact forces between particles by reducing each particle to a spheropolygon.

A model of this nature successfully identified several patterns of pedestrian movement, including the building of routes, the occurrence of landslides, and instances of blockages. The model was validated through extensive benchmark tests, including simulations of granular flow and many-body interactions, demonstrating that the spheropolygon method produces results consistent with theoretical expectations. The model's efficiency was further improved by implementing neighbor and contact lists, which reduced computational complexity and enabled large-scale simulations. These patterns are fundamental to understanding how pedestrians behave in a confined space [78]. Also, controlled experiments were carried out in a cylindrical controlled space to study contact forces between pedestrians in high-density situations. Real-time pressure data were captured using Tactilus visualization software by fixing the pressure sensors on the upper body surface of a pedestrian. The data are examined using Fisher-Shannon information metrics focusing on cyclic patterns and regularity of the contact forces for all different pedestrian densities [26]. Their study found that contact forces had a period of around 21 in sparser conditions, with values higher in danger conditions (SOR = 0.87).

Studies analyzed the spectral and information content of the force to which pedestrians are subjected under simulated congestion scenarios. Advanced methods of statistics applied to the analysis of properties in contact forces that enabled a better understanding of dynamic behaviors by pedestrians under crowd conditions. The results emphasized the cyclical nature of contact forces and the unique behaviors observed at varying crowd densities. A close study was carried out to investigate the interaction among pedestrians in high-density scenarios. The study used multiple methods of simulation to reproduce real-life crowd movements accurately. The methods employed in the (Alonso-Marroquin, 2008) [72] study involved sophisticated computational models to accurately measure the recreated physical forces generated by pedestrians. These modeling techniques make it possible to accurately determine and predict the contacting force with a small margin of error. Different numerical simulation methods have been used to study pedestrian dynamics, especially in measuring pedestrian contact forces. Developing new simulation methods and enhancing existing ones can lead to more accurate results, significantly contributing to research in pedestrian safety. Additionally, these advancements improve our understanding of crowd behavior and pave the way for better implementation of safety measures in high-density environments.

## 5 Future work outlook

To contribute meaningfully to the ongoing development of pedestrian dynamics research, it is crucial to outline a comprehensive set of future requirements and research directions to benefit the broader research community. Using AI to detect and analyze pedestrian behavior on contacting phenomena, there are other important challenges and opportunities under careful study. Understanding these issues will enrich our knowledge about pedestrian interactions in different environments and provide a foundation for more effective safety strategies in crowded areas. In this paper, we suggest two specific research directions.

(1) Sensor array-based investigation. While most studies have concentrated on measuring forces in the back, hands, and other body parts, the chest has rarely been explicitly mentioned as a focus, except in the study by Ref. [26]. Sensor arrays can enable detailed mapping of pressure distribution. Complex data analysis software can be utilized to process and represent the pressure data, which is particularly challenging when analyzing measurements from individual body parts. Therefore, we recommend a sensor-based investigation direction, incorporating more physical force sensors, including those for measuring forces on the chest or front body parts, in pedestrian crowded scenarios. This approach would provide a more comprehensive understanding of the forces in such conditions.
(2) Force measurement on slope micro-road networks. Most injuries and fatalities in pedestrian stampede accident occurred at the bottom of slopes and near micro-road networks in hospital settings. However, few studies have investigated these special areas. Most experiments have been conducted in controlled settings, such as flat areas, indoor corridors, staircases, or competitive evacuation drills, with minimal consideration given to sloped micro-road networks. To address this gap, ongoing experiments in our research group are focused on measuring pedestrian contact forces on a sloped micro-road using a novel model for force measurement. For instance, we suggest a model includes 12 sensors attached to different body parts: four sensors on the back, two on each shoulder, and four on the front body. Each sensor is connected to a controller, which can process data from up to 12 sensors and transmit pressure measurements to a smartphone via Bluetooth or Wifi communication.

**Fig. 7:**
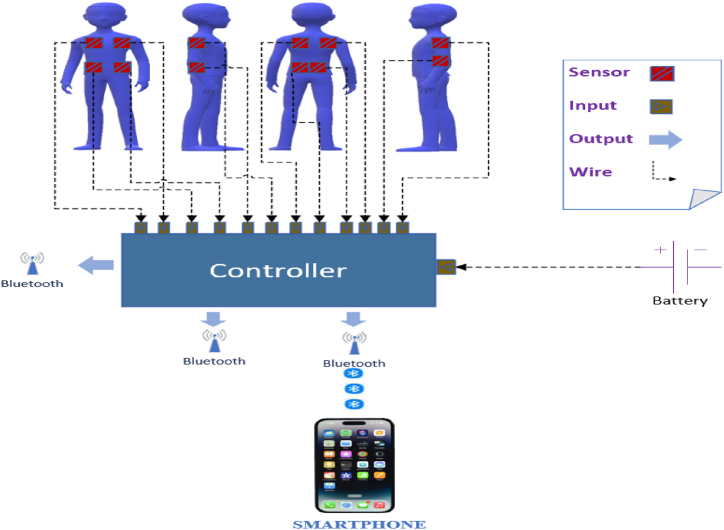
Sensor-based investigating diagram.

Fig. 7 shows an example outlook of a sensor-based investigating diagram. A small 5V 1500 mA battery powers the controller and the sensors. This cost-effective approach can enable rapid smartphone acquisition of force measurement results. This model is novel because that it is distinct from those used in previous research. More detailed information will be provided on upcoming studies. Measuring pedestrian contact forces with flexible pressure sensors has to address new challenges. For instance, most sensors are 40 × 40 mm in size and can measure forces ranging from 500g to 20 kg. While these sensors are small in size, they do have measurement limitations. The controller transmits data via Bluetooth, which restricts the range of data transfer. Additionally, the controller requires an external battery for power, which, though compact (66 x 55 × 40 mm, weighing 290g, with a 4–5 h battery life), may be cumbersome for volunteers during experiments. Implementing these sensors in real-world scenarios is further complicated by human participants' physical and psychological behaviors, particularly in high-density environments.

## 6 Conclusion

Pedestrian contacting force is critical in understanding pedestrian behavior under crowded conditions. In this article, the survey of different forces acting on pedestrians, such as pedestrian normal external forces, self-driven forces, abnormal external forces, and motion constraint forces from obstacles, has provided a significant understanding of the factors that influence pedestrian stability and movement. This review highlighted the influence of crowding posture on force distribution and demonstrated various pedestrian standing postures significantly impact the pedestrian crowding forces experienced by individuals. This perception is important for creating effective crowd management strategies and ensuring pedestrian safety in mass gathering areas.

We review researchers' current methodologies in measuring pedestrian contacting forces, including pressure measurement sensors, sophisticated motion-capturing systems, and advanced numerical simulations. These technologies have advanced the ability to measure and analyze pedestrian forces accurately; at the same time, this review identified a gap in research concerning slope micro-roads. Most studies have focused on flat areas, indoor corridors, staircases, and competitive evacuation drills, leaving a crucial area unexplored.

The proposed sensor-based model aims to measure the pressures exerted by pedestrians on a sloping micro-road network, covering the existing gap in knowledge. Attaching sensors on different body parts, including the back, front body, and shoulders, this model offers a complete approach to capturing the distribution of forces. The Bluetooth/Wifi-connected controllers and smartphone applications for real-time data analysis enhance the accessibility of this method. Further studies could build on the findings of this analysis by performing tests in different micro-road scenarios to verify the proposed structure. To reduce the risk of pedestrian crowd accidents and ensure the safety and security of those participating, it is necessary to integrate more AI technologies to promote pedestrian mechanics analysis and provide further theoretical support for safe pedestrian flow control.

## CRediT authorship contribution statement

**Rongyong Zhao:** Writing – review & editing, Supervision, Project administration, Methodology, Funding acquisition, Conceptualization. **Arifur Rahman:** Writing – original draft, Resources, Methodology, Formal analysis, Data curation, Conceptualization. **Bingyu Wei:** Visualization, Validation, Resources. **Cuiling Li:** Resources, Formal analysis. **Yunlong Ma:** Visualization, Validation. **Yuxing Cai:** Resources, Formal analysis. **Lingchen Han:** Resources, Formal analysis.

## Declaration of competing interest

The authors declare the following financial interests/personal relationships which may be considered as potential competing interests:Rongyong Zhao reports financial support was provided by 10.13039/501100001809National Natural Science Foundation of China. Rongyong Zhao reports a relationship with 10.13039/501100001809National Natural Science Foundation of China that includes: funding grants. If there are other authors, they declare that they have no known competing financial interests or personal relationships that could have appeared to influence the work reported in this paper.
